# Copy number variation analysis of m^6^A regulators identified METTL3 as a prognostic and immune‐related biomarker in bladder cancer

**DOI:** 10.1002/cam4.3981

**Published:** 2021-10-20

**Authors:** Xiaoshuai Wang, Jingwei Yu, Jinbao Chen, Yingdong Hou, Zefeng Du, Haoyang Huang, Siqi Tang, Yueyin Han, Changhai Ding, Zhicheng Xue

**Affiliations:** ^1^ Department of Gastric Surgery Sun Yat‐sen University Cancer Center, China State Key Laboratory of Oncology in South China Collaborative Innovation Center for Cancer Medicine Guangzhou China; ^2^ Clinical Research Centre Zhujiang Hospital Southern Medical University Guangzhou Guangdong China; ^3^ Department of Urology, Kidney and Urology Center Pelvic Floor Disorders Center The Seventh Affiliated Hospital Sun Yat‐sen University Shenzhen China; ^4^ Zhongshan Medical College of Sun Yat‐sen University Guangzhou China; ^5^ Department of Radiation Oncology Sun Yat‐sen University Cancer Center State Key Laboratory of Oncology in South China Collaborative Innovation Center for Cancer Medicine Guangdong Key Laboratory of Nasopharyngeal Carcinoma Diagnosis and Therapy Guangzhou China

**Keywords:** bladder cancer, copy number variation, immune cells, *N* ^6^‐methyladenosine, prognosis

## Abstract

**Purpose:**

Growing evidence has demonstrated an indispensable role for *N* ^6^‐methyladenosine (m^6^A) in human diseases, but the copy number variations (CNVs) of m^6^A regulatory genes in bladder cancer (BLCA) remains largely unknown.

**Methods:**

We investigated the CNVs on all known m^6^A regulatory genes using the Cancer Genome Atlas (TCGA) database. The association between CNV events and clinicopathological as well as molecular characteristics of BLCA patients were explored. Gene set enrichment analysis (GSEA) was implemented to reveal relative cellular processes. Association between m^6^A regulatory genes and immune infiltrates was analyzed by The Tumor Immune Estimation Resource (TIMER) database.

**Results:**

CNV events of m^6^A regulatory genes were frequently observed in BLCA. CNVs of METTL3, METTL14, and METTL16 correlated with molecular characteristics of BLCA patients including TP53 mutation. CNVs of METTL3 associated with the overall survival (OS) of BLCA patients. METTL3 was also associated with several cancer‐related cellular processes, including mitotic spindle assembly, G2/M checkpoint, and E2F targets signaling pathway. Besides, the CNVs of m^6^A regulatory genes were correlated with specific kinds of immune infiltrates.

**Conclusions:**

There are significant correlations between m^6^A regulatory genes with CNVs and clinicopathological characteristics. METTL3 with CNVs were associated with the immune infiltrates and performed as a prognostic marker in BLCA.

## Graphical abstract

We investigated the CNV events on all known m6A regulatory genes which were obtained from the Cancer Genome Atlas (TCGA) database through bioinformatics analysis of prognosis as well as immune infiltrates of BLCA patients. We found that CNVs of METTL3 associated with the overall survival (OS). Furthermore, RNA methyltransferases may affect the outcome of tumor immunotherapy via regulating immune infiltrates.

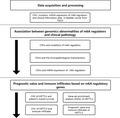

## 1 INTRODUCTION

Bladder cancer (BLCA) is one of the most common tumors with high morbidity and mortality worldwide.^1^ BLCA is the eighth most frequently diagnosed cancer in men in 2018, accounting for approximately 4% of all cancer‐related deaths in the United States.^2^ There are two major classifications of BLCA. According to histological features, BLCA is classified as urothelial carcinomas, squamous cell carcinoma, small‐cell carcinoma, and adenocarcinoma.^3^ Urothelial carcinomas account for approximately 90% of all diagnosed patients.^4^ There is another classification according to whether the tumors invade the detrusor muscle. They are classified as non‐muscle‐invasive bladder cancer (NMIBC, approximately 75%) and muscle‐invasive bladder cancer (MIBC, approximately 25%).^5^ The latter classification is widely used in clinical practice relatively ^1^ since the prognosis of two subtypes varies greatly. Usually, NMIBC is treated with transurethral resection, and followed by intravesical Bacillus Calmette‐Guerin (BCG) or intravesical chemotherapy. While MIBC is typically treated with radical cystectomy and neoadjuvant chemotherapy because of higher rates of progression and recurrence.^6^ Despite the progress in surgical techniques and adjuvant therapy, the 5‐year survival rate of bladder cancer with metastasis is about 8%.^7^ Last decade, immunotherapy had been proved to be a favorable regimen for both early and late stages in BLCA.^8^, ^9^ For example, Nivolumab, an immunotherapy drug targeting programmed cell death protein 1 (PD‐1), was confirmed to induce neoplastic cell death in urothelial bladder cancers with metastases.^10^ Therefore, exploring immunotherapeutic target is promising for the diagnosis as well as treatment of BLCA.

The genetic and epigenetic alterations of DNA, such as gene mutations and copy number variations, are frequently reported in BLCA.^11^ It has been reported that mutations of specific genes like *TP53* may lead to tumor progression by dysregulation of cell cycle and DNA damage response.^12^ Copy number variations (CNVs) of several genes like *CCNE1* were also reported to have good diagnostic and prognostic value.^13^ Apart from these alterations, emerging evidence has revealed that RNA modifications are important for post‐transcriptional regulation of gene expression. ^14^ *N* ^6^‐methyladenosine (m^6^A) modification is one of the most common RNA modifications in mammalian systems.^15^, ^16^ It is regulated dynamically by methyltransferases (*METTL3*, *METTL14*, *METTL16*, *WTAP*, *RBM15*, *ZC3H13*, and *KIAA1429*), binding proteins (*YTHDC1*, *YTHDC2*, *YTHDF1*, *YTHDF2*, and *HNRNPC*) and demethylases (*ALKBH5* and *FTO*). Recent studies indicated that among these regulators, RNA methyltransferases (*METTL3*, *METTL14*, and *METTL16*) play indispensable roles in progression of BLCA. For instance, *METTL3* promotes the expression of *ITGA6*, resulting in increased growth and progression of BLCA,^17^ while *METTL14* inhibits bladder tumorigenesis by reducing mRNA stability of *NOTCH1*.^18^ It was indicated that m6A RNA methylation regulators contributed to the malignant progression of BLCA.^19^ Jin et al reported that m6A modification of ITGA6 promoted the development and progression of BLCA.^17^ Furthermore, CNVs were enriched in specific molecular subtypes of BLCA.^11^ While some novel molecular subtypes of BLCA were found based on immune‐cell‐associated CpG sites.^20^ Elizabeth et al found that copy number variations of FGFR‐3 were negatively correlated with Fibroblast growth factor 2 (FGF‐2) while positively associated with NMIBC.^21^ Recent studies also revealed the significantly association between tumorigenesis and copy number variations of m6A‐related genes in head and neck squamous cell carcinoma (HNSCC)^22^ and clear cell renal cell carcinoma (ccRCC).^23^ However, the copy number variations, expression and prognostic value of m^6^A regulators in bladder cancer remain largely unknown.

A growing amount of evidence reveals that immune infiltrate plays an essential role in many kinds of tumors and correlates with the effect of tumor immunotherapy.^24^, ^25^ Immune infiltrate is a part of the complex tumor microenvironment.^26^ A recent study has reported that tumor‐infiltrating immune cells are prognostic factors of lung cancer, and the infiltration status is also correlated with expressions of specific m^6^A regulatory genes including *METTL3*.^27^ Tumor‐infiltrating immune cells (TIICs) are composed of T cells, macrophages, dendritic cells, neutrophils, and mast cells. BLCA is an immune sensitive tumor infiltrated by TIICs.^28^ Two most widely applied immunotherapies in BLCA are Bacillus Calmette–Guerin (BCG) intravesical instillation and anti‐PD‐1/PD‐L1 immune checkpoint blockade.^29^

It should be noted that m6A methylation majorly regulated immune response in tumor immune microenvironment (TIME). For example, the low level of m6A increased infiltration of immune cells in the TIME, thus enhancing antitumor immunity and sensitivity to anti PDL‐1 immunotherapy in gastric cancer.^30^ However, the associations between the CNVs of m6A regulators and immune infiltrates in BLCA were yet to be clarified.

In this work, we systematically analyzed the alterations of m^6^A RNA methylation regulators in 409 bladder cancer from the Cancer Genome Atlas (TCGA) database. The association between genetic alterations of m^6^A regulators and clinicopathological characteristics of the bladder cancer cohort was investigated. We also identified the associated pathways in bladder cancer. Finally, the correlations between copy number variations and tumor infiltration levels were computed. Generally, we provided evidence that m^6^A regulators might play crucial roles in bladder cancer and serve as potential prognostic biomarkers.

## 2 MATERIALS AND METHODS

### 2.1 Data processing

The mRNA expression and clinical information were downloaded from the TCGA database (http://cancergenome.nih.gov/), which was based on Illumina HiSeq RNA‐Seq platform. The histological types of BLCA containing urothelial carcinomas, squamous cell carcinoma, small‐cell carcinoma, and adenocarcinoma were applied in further analysis. Only 363 patients had CNV data and all of them were included for further analysis. The mutation data of m^6^A regulators were obtained from the TCGA program by cBioportal platform,^31^ and a total of 87 patients were found to have mutation data.

### 2.2 Selection of m^6^A methylation regulators

First, we made a list of m^6^A regulators from published studies. Totally sixteen regulators were found.^16^, ^32^, ^33^ Due to lack of CNV data, only thirteen representative genes were finally selected with CNV. Second, we compared the CNV events of these regulators in BLCA with the clinicopathological features. The data of frequently mutated gene in BLCA was provided by Department of Urology, the Seventh Affiliated Hospital of Sun Yat‐sen University.

### 2.3 Bioinformatics analysis

To clarify the functions of m6A RNA methylation regulators in BLCA, we performed a gene set enrichment analysis (GSEA) under the condition of high expression METTL3 (defined as the mRNA level of METTL3 higher than the median level) and low expression METTL3 (defined as the mRNA level of METTL3 lower than the median level).^34^, ^35^ GSEA was provided by the JAVA program with MSigDB v6.1, which was downloaded from the Broad Institute.^36^ Hallmark gene set “h.all.v6.0.symbols.gmt” was used in this study.^37^ Gene sets with normalized *p*‐value <0.05, and the false discovery rate (FDR) <0.25 were considered to be significantly enriched.^23^, ^38^

### 2.4 Immune infiltrate signature analysis

The correlations between copy number variations and tumor infiltration levels were generated by the Tumor Immune Estimation Resource (TIMER).^39^ We used box plots to show the distributions of each immune subset at each copy number status in specific cancer types. The infiltration level for each category is compared with the normal, and two‐sided Wilcoxon rank sum test was used for analysis. The correlations between expression levels and the infiltration levels of six cell types (B cells, CD4^+^ T cells, CD8^+^ T cells, neutrophils, macrophages, dendritic cells) were computed,^40^ and the results were generated by TIMER.

### 2.5 Statistical analysis

One‐way ANOVA was used to compare the expression level of m^6^A regulators with different CNV patterns in BLCA. Chi‐square test was applied to describe the association between CNV of m^6^A regulators and clinical characteristics and the association between CNV patterns and typical genes mutation in BLCA. Kaplan–Meier method with a two‐sided log‐rank test was used to compare the OS of BLCA patients in the deletion‐ and gain‐risk groups. Cox proportional hazards regression analysis was used to identify the relationship between m6A regulatory genes and BLCA patients’ OS. Results with a *p*‐value <0.05 were considered significant. All statistical analyses were performed using R v3.6.1 (https://www.r‐project.org/) and Prism 8.0.1 (GraphPad Software Inc.).

## 3 RESULT

### 3.1 Mutations and CNVs of m6A regulators in BLCA patients

**FIGURE 1:**
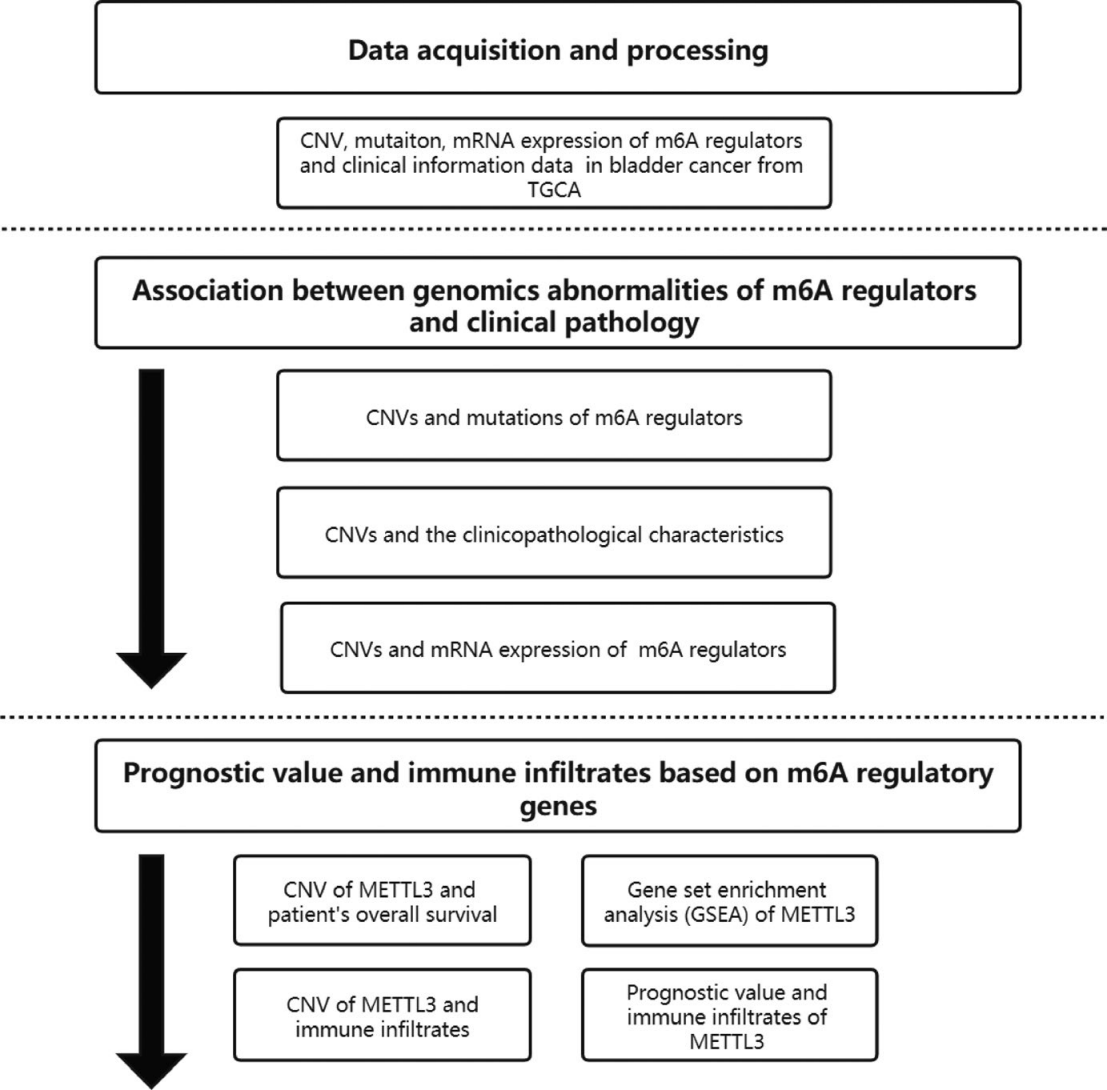
The flowchart of the study design

**FIGURE 2:**
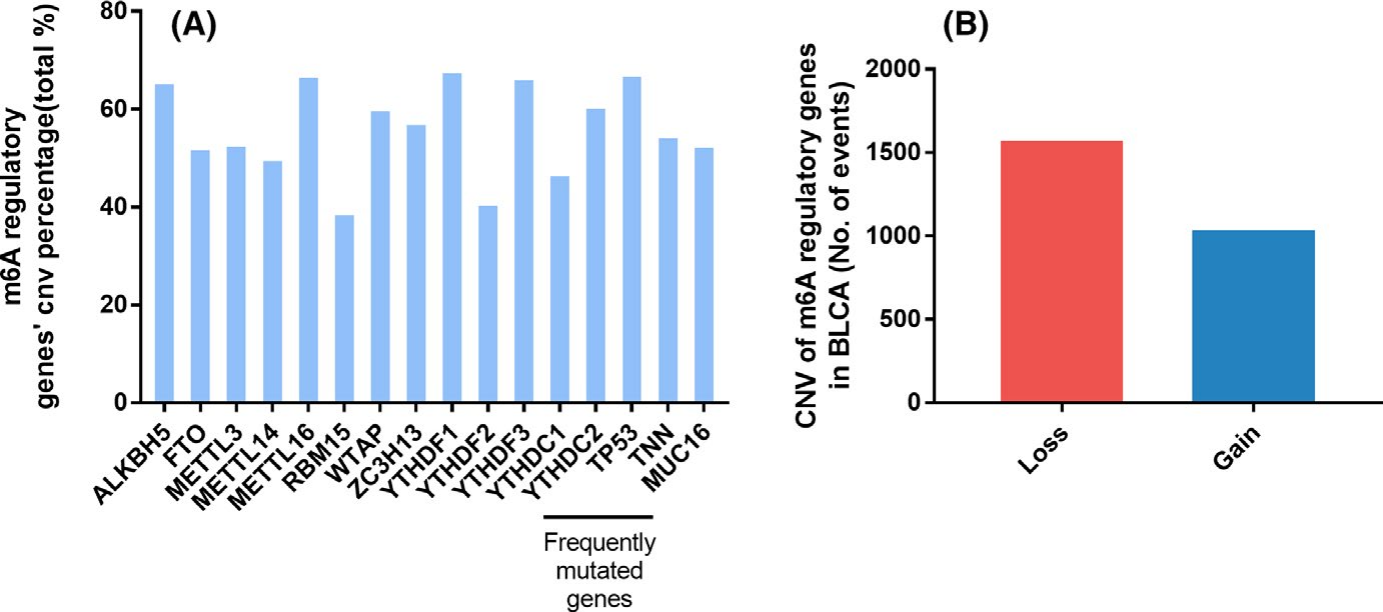
CNVs of m6A regulatory genes and three genes that were frequently mutated in BLCA. (A) Percentage of BLCA samples with CNV events of the m6A regulatory genes. (B) CNV events of copy number gain and loss in BLCA samples

A flowchart of the study design is shown in Figure 1. Considering m6A regulators played important role in bladder cancer, we performed a comprehensive bioinformatics analysis to explore the relationships between genomics abnormalities of m^6^A regulators and clinical characteristics of BLCA based on the TCGA database. Among the 87 patients who have mutation data obtained from cBioportal platform, mutations of the thirteen m^6^A regulatory genes were seldom detected (Table 1). However, CNV events of m^6^A regulators were frequently observed in 363 samples of BLCA with CNV data in TCGA database (Figure 2 and Table 2). Among three RNA methyltransferases, CNVs of *METTL3* (192/363, 52.89%) and *METTL16* (243/363, 66.94%) were found in more than half of the samples and those of *METTL14* (181/363, 49.86%) in nearly half of the samples. Among these 363 samples, most of the CNV events of m^6^A regulatory genes led to a loss of copy number (1586/2635).

**TABLE 1:** Mutations count of m^6^A regulatory genes in 87 BLCA patients

M^6^A regulatory genes	Mutations count
METTL3	18
METTL14	5
METTL16	1
WTAP	7
RBM15	14
ZC3H13	16
FTO	4
ALKBH5	5
YTHDF1	4
YTHDF2	9
YTHDF3	3
YTHDC1	8
YTHDC2	13

Mutation count represented the number of patients with corresponding gene mutations.

**TABLE 2:** Different CNV patterns of m^6^A regulatory genes and three frequently mutated genes occurred in BLCA samples (n = 363)

	Gene	Diploid	Deep deletion	Shallow deletion	Copy number gain	Amplification	CNV sum	Percentage
Eraser	ALKBH5	125	9	177	49	3	238	65.56%
	FTO	174	3	115	69	2	189	52.07%
Writer	METTL3	171	4	110	73	5	192	52.89%
	METTL14	182	1	147	30	3	181	49.86%
	METTL16	120	7	190	45	1	243	66.94%
	RBM15	222	1	59	76	5	141	38.84%
	WTAP	145	4	181	33	0	218	60.06%
	ZC3H13	155	20	118	68	2	208	57.30%
Reader	YTHDF1	117	0	23	220	3	246	67.77%
	YTHDF2	215	0	79	66	3	148	40.77%
	YTHDF3	122	0	28	200	13	241	66.39%
	YTHDC1	193	1	116	46	7	170	46.83%
	YTHDC2	143	6	187	25	2	220	60.61%
Mutated genes	TP53	119	10	192	41	1	244	67.22%
	TNN	165	1	26	149	22	198	54.55%
	MUC16	172	1	121	65	4	191	52.62%

### 3.2 Association between CNV events of m^6^A regulators and clinicopathological as well as molecular characteristics

**FIGURE 3:**
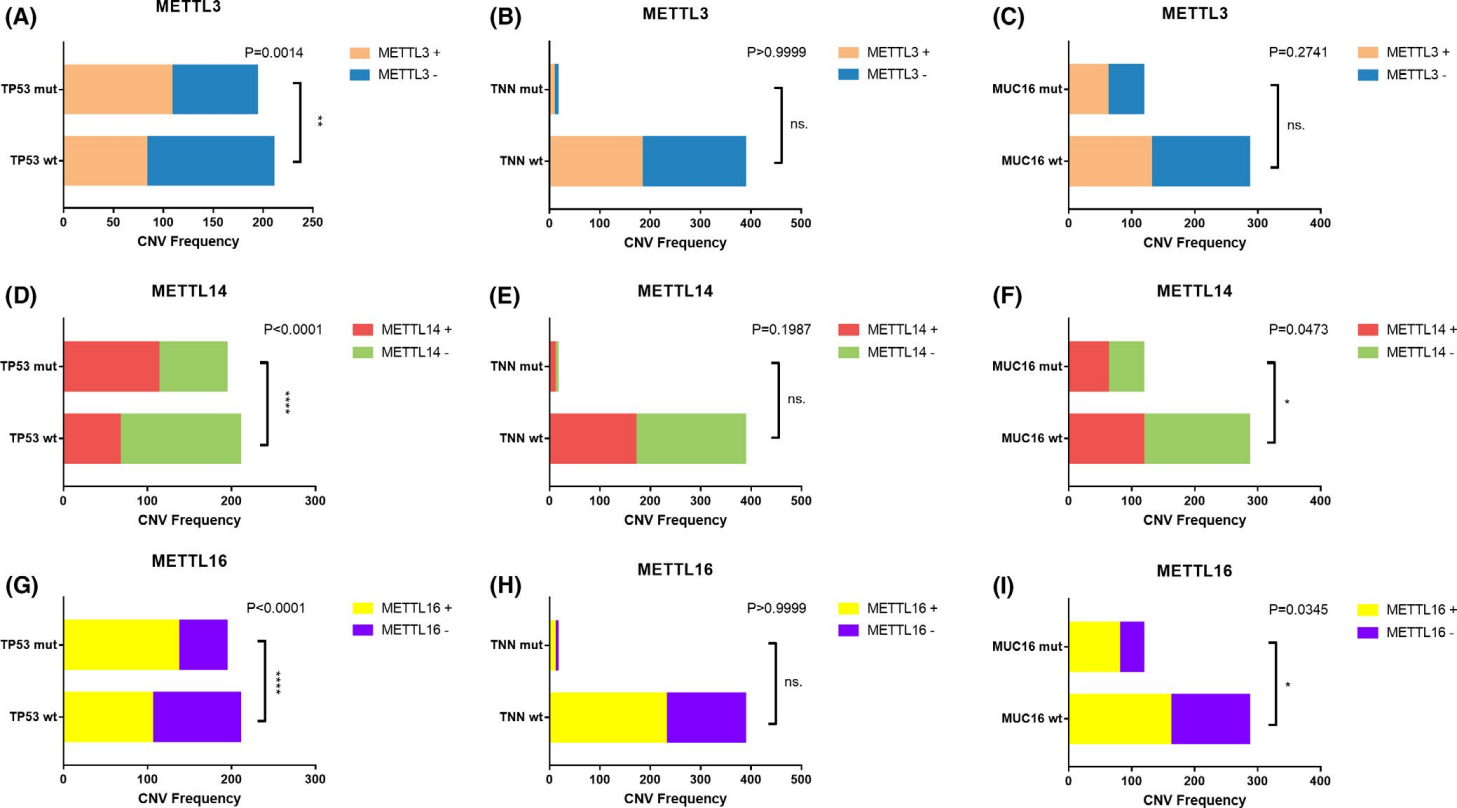
Relationship between molecular characteristics and CNVs in (A‐C) METTL3, (D‐F) METTL4, and (G‐I) METTL16, respectively. Pearson Chi‐square test was applied for analysis, and *p* < 0.05 was considered significant. METTL3+ METTL3 with CNVs, METTL3‐ METTL3 without CNVs, METTL14+ METTL14 with CNVs, METTL14‐ METTL14 without CNVs, METTL16+ METTL16 with CNVs, METTL16‐ METTL16 without CNVs. *p < 0.05, **p < 0.01, ***p < 0.001, ****p < 0.0001, ns. not significant

We previously investigated the most frequently mutated genes in bladder cancer in our center, and the top 3 of these genes (*TP53*, *TNN*, *MUC16*) were used for analysis (Table S1). We found that the presence of CNV events of m^6^A regulatory genes, especially CNV events of *METTL3*, *METTL14*, and *METTL16* were significantly correlated with mutations of *TP53* (Figure 3 and Table 3). The samples were also divided into two groups: one consisted of samples with mutations and/or CNVs of m^6^A regulatory genes while the other consisted of samples without mutations or CNVs. The relationship between these two groups and the clinicopathological characteristics of BLCA patients was assessed. Advanced age (*p* = 0.019), pathological TNM stage (*p* = 0.026), pathological N stage (*p* = 0.02) and histological grade. (*p* < 0.001) of BLCA were found significantly related to the occurrence of mutations and/or CNV events (Table 4).

**TABLE 3:** Relationship between molecular characteristics and CNV events of m^6^A regulatory genes in BLCA patients (n = 409)

			Without mutation nor CNV*	With mutation and/or CNV*	χ²	***p***
TP53	n = 409	wt	35	177	20.552	<0.001
		alteration	6	191		
TNN	n = 409	wt	32	181	12.315	<0.001
		alteration	9	187		
MUC16	n = 409	wt	37	254	8.094	0.004
		alteration	4	114		

Chi‐square test was applied for analysis and *p* < 0.05 was considered significant.

* With mutation and/or CNV: Cases have mutant or CNV or mutant and CNV, confirmed through TCGA database. Without mutant nor CNV: Cases with neither mutant nor CNV, confirmed through TCGA database. Ambiguous variables (Nx, Mx, N/A and Gx) were excluded from chi‐square test or non‐parametric test.

**TABLE 4:** Clinical pathological parameters of BLCA patients with or without mutation or CNV of m^6^A regulatory genes

		With mutation and/or CNV*	Without mutation nor CNV*	*p*
Age	<=60	90	17	0.019
	>60	278	24	
Gender	Female	97	9	0.541
	Male	271	32	
Pathological TNM Stages	Ⅰ	1	1	0.026
	Ⅱ	112	18	
	Ⅲ	124	15	
	Ⅳ	129	7	
	N/A	2	0	
T stage	T1	2	1	0.125
	T2	103	15	
	T3	174	18	
	T4	56	2	
	Tx	29	3	
M stage	M0	166	27	0.993
	M1	10	1	
	Mx	187	11	
N stage	N0	204	31	0.02
	N1	44	1	
	N2	73	3	
	N3	7	0	
	Nx	33	3	
Histological Grade	low	13	8	<0.001
	high	352	33	
	N/A	3	0	

A chi‐square test was applied for analysis and *p* < 0.05 was considered significant.

* With mutation and/or CNV: Cases have mutant or CNV or mutant and CNV, confirmed through TCGA database. Without mutant nor CNV: Cases with neither mutant nor CNV, confirmed through TCGA database. Ambiguous variables (Nx, Mx, N/A and Gx) were excluded from chi‐square test or non‐parametric test.

**FIGURE 4:**
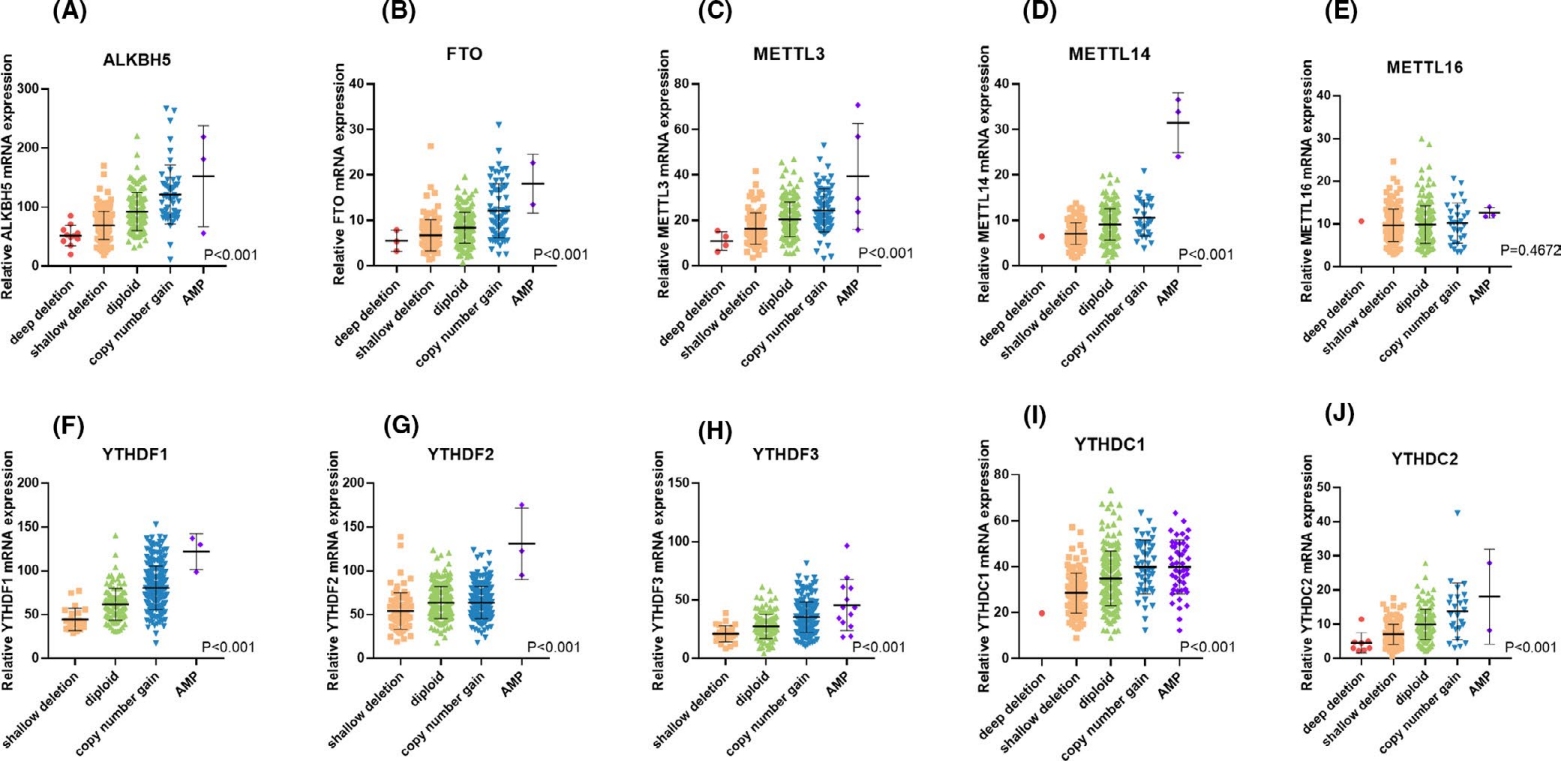
Correlation between different CNV events (deep deletion, shallow deletion, diploid, copy number gain, AMP) of m6A regulatory genes mRNA expression levels (A–J). One‐way ANOVA was applied to determine the significance, and *p* < 0.05 was considered significant

The most mutation counts were merely 18 (Table 1), which indicated the few mutations of BLCA.^23^ Furthermore, the occurrence of mutations and/or CNVs of m^6^A regulatory genes were significantly associated with the prognosis of BLCA (Table 4). Thus, we paid more attention to whether CNVs contributed to the change of m^6^A level in BLCA. We next evaluated the effect of CNV events on mRNA expression of m^6^A regulatory genes. Among all the regulatory genes, CNV events of most genes were significantly correlated with their mRNA expression, respectively (Figure 4 and Figure S1). Copy number gain and amplification (AMP) were associated with higher expression levels, while deep deletion and shallow deletion led to lower expression levels.

### 3.3 CNVs of METTL3 associated with overall survival of BLCA patients

**FIGURE 5:**
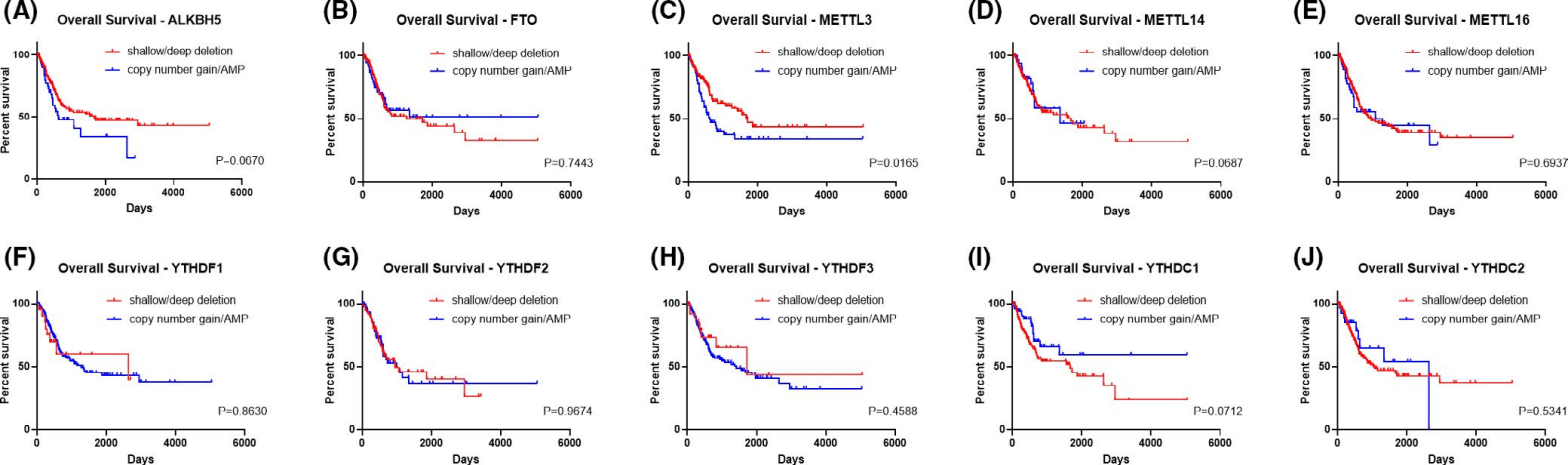
Overall survival of BLCA patients with different CNV types (deep deletion, shallow deletion, diploid, copy number gain, AMP) of m6A regulatory genes (A–J). Kaplan‐Meier method was used for analyses and *p* < 0.05 was considered significant

Survival analysis was then performed to explore the prognostic value of CNVs in m^6^A regulators among BLCA patients. A separate analysis of these ten genes revealed that only CNVs in *METTL3* were significantly correlated with the OS of the BLCA cohort. Patients with copy number gain and AMP of *METTL3* had an inferior OS (Figure 5 and Figure S2). However, no significant correlation was found between OS and CNVs of either *METTL14* or *METTL16*. Further univariate Cox regression analyses revealed that CNVs of *METTL3* was related to OS, similar to other clinical covariates such as age and tumor grade. However, none of these variates was independent factors of survival status of BLCA patients according to the result of multivariate Cox regression (Table 5).

**TABLE 5:** Univariate and multivariate Cox regression analysis of different factors in BLCA patients

Variables	Univariate analysis		Multivariate analysis	
	HR (95% CI)	*p*	HR (95% CI)	*p*
Age (>60 vs <=60)	2.107 (1.374–3.231)	**0.001**		0.824
Tumor grade (I‐II vs III‐IV)	2.681 (1.758–4.087)	**<0.001**		0.897
M (M1 vs M0)	3.018 (1.372–6.639)	**0.006**		0.025
N (N1 vs N0)	2.134 (1.350–3.372)	**0.001**		0.829
T (T3‐T4 vs T1‐T2)	2.438 (1.603–3.708)	**<0.001**		0.982
METTL3 (copy number gain/AMP vs shallow/deep deletion)	1.712 (1.097–2.672)	**0.018**		0.331

*p* < 0.05 was considered significant.

### 3.4 Gene set enrichment analysis (GSEA) of METTL3

**FIGURE 6:**
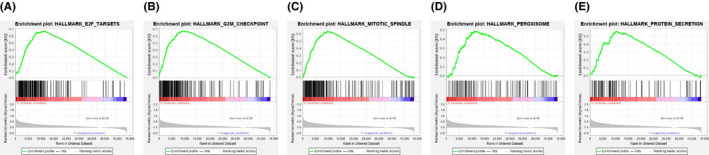
GSEA results of different expression level of METTL3. Gene set enrichment plots of (A) E2F targets, (B) G2‐M checkpoint, (C) mitotic spindle, (D) peroxisome, and (E) protein secretion were shown

Given the significance of *METTL3* in the m^6^A modification and the surprising results we found, we explored the effect of dysregulated m^6^A on the pathogenesis of BLCA. GSEA was performed to identify the gene sets enriched in these samples with different mRNA expression levels of *METTL3*. We found that high expression level of *METTL3* associated with several cancer‐related biological processes, including mitotic spindle formation, G2‐M checkpoint signaling, and E2F targets signaling pathway (Figure 6 and Table S2). High expression of *METTL14* was also correlated with several pathways, and the results were showed in Table S3.

### 3.5 METTL3 associated with the immune infiltrates in BLCA

**FIGURE 7:**
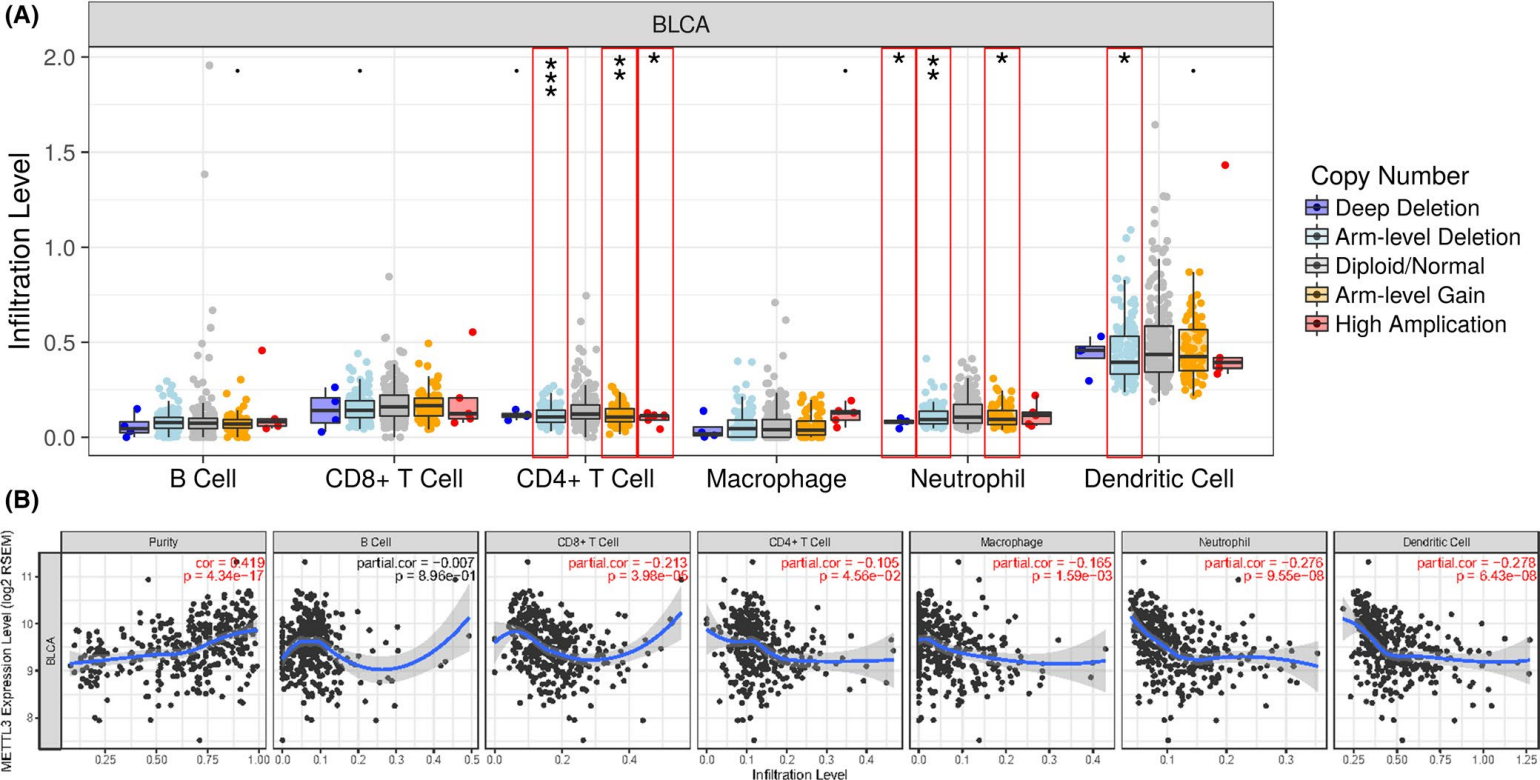
TIMER revealed that both CNV events and expression of METTL3 were associated with immune infiltrates. (A) CNVs of METTL3 were significantly correlated with immune infiltrates of CD4^+^ T cells, Neutrophils and Dendritic cells. *p < 0.05, **p < 0.01, ***p < 0.001. (B) METTL3 expression was significantly associated with almost all kinds of immune infiltrates. *p* < 0.05 was considered significant and highlighted in red

We finally computed the infiltration levels of different immune cell types including B cells, CD4^+^ T cells, CD8^+^ T cells, neutrophils, macrophages, and dendritic cells. The results highlighted the correlation between RNA methyltransferase and immune infiltrates (Figure 7 and Figure S3). The CNV events of both *METTL3* were significantly correlated with the downregulated infiltration levels of CD4^+^ T cells, neutrophils, and dendritic cells. On the other hand, the expression level of *METTL3* was significantly associated with most kinds of immune infiltrates. The correlation between *METTL14* and immune infiltrates was also computed and showed in Figure S3.

## 4 DISCUSSION

In this study, we discovered high frequency of CNV events of m^6^A regulatory genes in BLCA, which were associated with clinicopathological as well as molecular characteristics, the prognosis, oncogenic signaling pathways, and immune infiltrates.

*N^6^*‐methyladenosine (m^6^A) is one of the most abundant internal RNA modifications in mammalian systems.^41^ In recent years, a number of studies chose to evaluate the altered expression of m^6^A regulatory genes to portrait a unique expression pattern of specific pathological process, indirectly exploring the m^6^A level and biological significance in human diseases. Because of the distinct m^6^A status of different kinds of tumors, we aimed to explore the CNV events of m^6^A “writers”, “erasers” and “readers”. In BLCA cohort, the frequency of CNV events of thirteen m^6^A regulatory genes was higher than that reported in ccRCC^23^ and AML,^32^ especially m^6^A “writers”, suggesting that m^6^A might play a more indispensable role in BLCA. Furthermore, CNV data showed that all three kinds of m^6^A regulatory genes had a high frequency of CNV events, indicating that the regulation of m^6^A modification in BLCA was so complicated that further investigations are needed to demonstrate the m^6^A regulatory mechanism in BLCA.

After a general understanding of CNV events in BLCA patients, we evaluated the association between alterations of m^6^A regulators and clinicopathological and molecular characteristics of BLCA cohort. Our results showed that alterations of these genes were significantly related to the age of patients, pathological TNM stage, and histological grade of BLCA. These results suggested that CNV alterations may associate with histology transformation, similar to ccRCC,^23^ making it possible for CNV alterations of these genes to serve as biomarkers predicting the grade of bladder cancer. Furthermore, alterations of m^6^A regulators were found significantly correlated with alterations of *TP53*, *TNN*, and *MUC16*, which were frequently detected as mutated genes,^42^ similar to the results of AML. *TP53* was the most frequently altered genes among these three genes. And most of the CNVs led to a loss of copy number (shallow/deep deletion: copy number gain/AMP = 202:42).^32^ It was also reported that altering the expression of *METTL3* would significantly affect the expression level and alternative splicing patterns of certain genes, resulting in modulation of downstream targets of the *TP53* signaling pathway such as *P21*, *FAS*, and *BAX*.^43^ These effects would lead to inhibition of apoptosis and tumorigenesis. Together, these findings suggested that alterations of m^6^A regulatory genes and *TP53* signaling pathway may synergistically play important roles in BLCA pathogenesis and progression.

In our study, *METTL3* showed slightly increase in deletion than copy number gain and amplification. However, recent studies had demonstrated that expression level of *METTL3* was significantly upregulated in BLCA.^44^, ^45^ It has been proven that *METTL3* could promote BLCA progression via *AFF4*/*NF*‐*κb*/*MYC* signaling network.^44^ *METTL3* was also shown capable of promoting BLCA proliferation by accelerating pri‐*miR221*/*222* maturation in an m^6^A‐dependent manner.^45^ These findings suggested that although a significant association between CNV patterns and mRNA expression levels was found in most of the m^6^A regulatory genes including *METTL3*, the expression levels of these genes may not be directly predicted by CNV patterns. Still, further studies on regulation mechanisms between CNV and gene expression and the following functions are needed to clarify the relationship between CNV and human diseases.

The effect of CNV events of m^6^A regulatory genes on the survival of BLCA patients was also evaluated. Among all these regulatory genes, only the CNVs on *METTL3* were significantly associated with the OS of BLCA patients. It is noteworthy that although most of its CNVs led to a loss of copy number (shallow/deep deletion: copy number gain/AMP = 114:78), copy number gain and AMP of *METTL3* were linked to poor OS. Despite the fact that *METTL3* and other clinical covariates such as age, pathological TNM stage were associated with OS according to the results of univariate analysis, none of them showed the consistent result in multivariate analysis. However, we should still attach importance to the effect of *METTL3* alteration on survival as prior studies have established that alteration of *METTL3* could promote tumor proliferation and migration, which are related to higher T stage and tumor grade of BLCA.^17^, ^44^ Further analyses of regulation mechanisms of *METTL3* alterations on other clinical covariates are needed to illustrate this contradictory phenomenon.

Several cancer‐related biological processes were found to be dysregulated in BLCA. We found in this BLCA cohort that downregulated expression of *METTL3* was associated with some pathways including mitotic spindle assembly, G2‐M checkpoint signaling and E2F targets signaling pathway, which are all important cellular processes in tumorigenesis.^46^, ^47^ These findings suggest that these processes may also play indispensable roles in BLCA pathogenesis, and it is likely that alterations of m^6^A regulatory genes can regulate BLCA progression via these pathways. However, the specific regulation mechanisms of *METTL3* alteration on these pathways is yet to be clarified.

Given the latest finding of the association between m^6^A regulatory genes and immune infiltrates,^27^ we finally computed that both CNV events and expression of *METTL3* were negatively correlated with immune infiltration levels of CD4^+^ T cells, neutrophils, and dendritic cells. These findings were also partly confirmed in lung squamous cell carcinoma.^27^ Previous studies have established that immune infiltrates in BLCA were composed mainly by CD4^+^ T cells,^48^ and the infiltration level was negatively correlated with BLCA stage and risk of recurrence.^49^ Under some aspects of cell pathology including tumorigenesis, several signaling pathways would be activated for immune cell recruitment, such as *CCL2*/*CCR2* pathway^50^ and *CCL5*‐related pathway.^51^ Collectively, these findings suggested that RNA methyltransferases may regulate BLCA immune infiltrates in an m^6^A‐dependent manner via specific signaling pathways, and CD4^+^ T cells could be the main targets. However, the relationship between m^6^A and immune infiltrates was hardly reported, and these results highlighted the need for more in‐depth studies.

Recent studies indicated that METTL3 promoted the expression of ITGA6, resulting in the growth and progression of BLCA.^17^ In our study, we found that CNVs of METTL3 were significantly correlated with the OS of the BLCA cohort. Besides, CNVs of METTL3 were essentially associated with the immune infiltrates in BLCA. The CNV events of METTL3 were significantly correlated with the downregulated infiltration levels of CD4^+^ T cells, neutrophils, and dendritic cells. Furthermore, CD4^+^ T cells that highly expressed the forkhead box P3 (FOXP3) suppressed the anti‐tumor immunity and suggested a poorer prognosis in colorectal cancers.^52^ While the high infiltration level of neutrophils was also associated with a poorer prognosis in most tumors.^53^ Moreover, it suggested that the immunoregulation of dendritic cells might be a prognostic indicator in breast cancer ^54^ Besides, METTL3‐mediated mRNA m^6^A methylation was associated with the immunocompetence of neutrophils^55^ and dendritic cells.^56^ Hence, we proposed that METTL3 might affect the prognosis of BLCA by regulating the immune infiltration level, which indicated that METTL3 might be a target to regulate the immune response of BLCA. In addition, we found significant correlations between CNVs of m^6^A regulatory genes and mutations of TP53. And TP53 mutations in tumors leading to enhanced expression of cell cycle progression genes and proteins was related a poor prognosis.^57^ Hence, we speculated that m^6^A regulatory genes and signaling pathways involving TP53 may synergistically contribute to BLCA pathogenesis and progression. It suggested that the CNVs of m^6^A regulators might be an early diagnostic indicator in the future. Moreover, Shi et al.’s study^58^ reported that patients with CNVs of m^6^A regulators genes were significantly associated with inferior OS in non‐small cell lung cancer, which was consistent with our findings in BLCA. Since the significant correlation between METTL3 and OS in BLCA patients, it indicated that CNVs of METTL3 might perform as a prognostic marker for BLCA. However, further mechanism on CNVs of METTL3 was yet to be explored.

## 5 CONCLUSION

There are significant correlations between m^6^A regulatory genes with CNVs and clinicopathological characteristics. METTL3 with CNVs were associated with the immune infiltrates and performed as a prognostic marker in BLCA.

## CONFLICTS OF INTERESTS

The authors declare no conflict of interests.

## AUTHORS’ CONTRIBUTIONS

Conceptualization: DCH and XZC; Data curation: WXS, YJW and HYD; Formal analysis: WXS, CJB HYD, and DZF; Investigation: WXS, CJB, HYD, and TSQ; Methodology: WXS, DZF, and HHY; Resources: CJB, TSQ, and HYY; Software: WXS and HYD; Supervision: WXS, DCH, and XZC; Validation: WXS and CJB; Visualization: DZF, TSQ and HYY; Writing—original draft, WXS and YJW; Writing—review and editing, WXS, DCH, and XZC. All authors have read and approved the manuscript.

## Supporting information

Fig S1

Fig S2

Fig S3

Table S1‐S3
