# Supplementary figures and images for: Copy number variation analysis of m6A regulators identified METTL3 as a prognostic and immune‐related biomarker in bladder cancer
Source: Cancer Med. 2021 Oct 20;10(21):7804–15. doi: 10.1002/cam4.3981 (PMC8559456; doi:10.1002/cam4.3981)

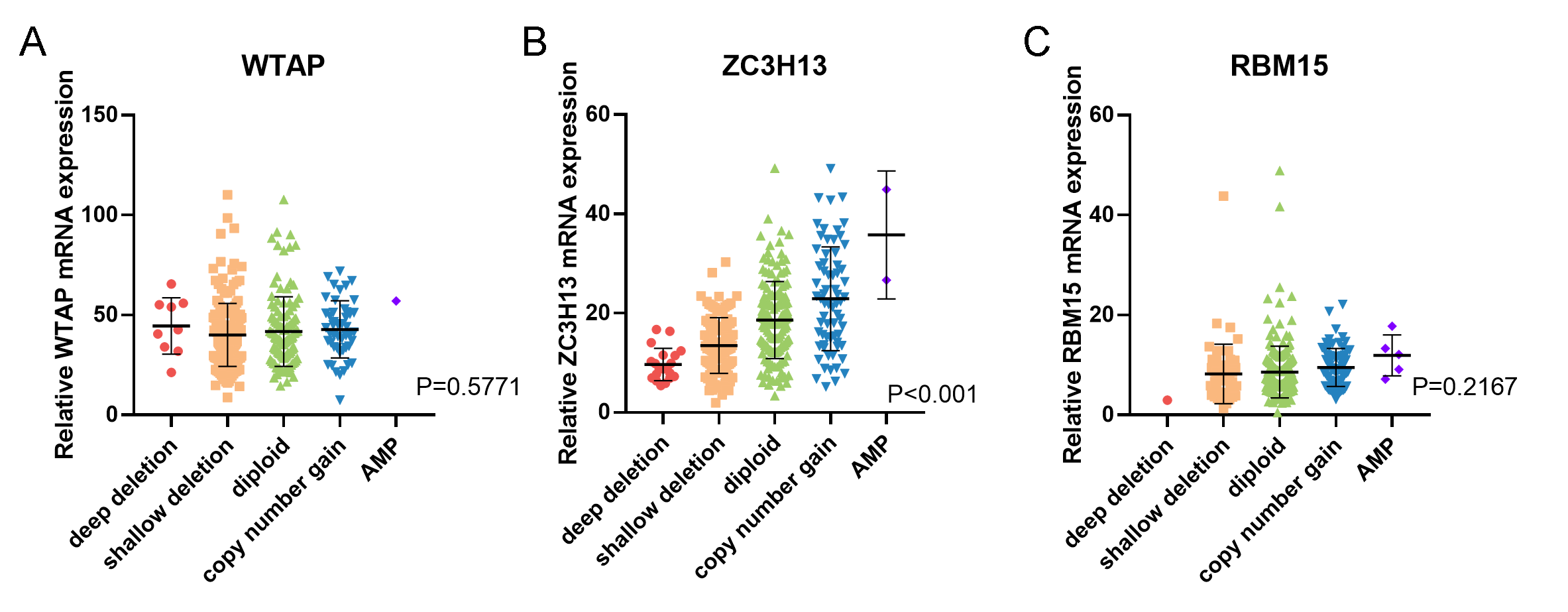

Supplement: Supplementary file 1 — Fig S1 [file CAM4-10-7804-s002.tif]

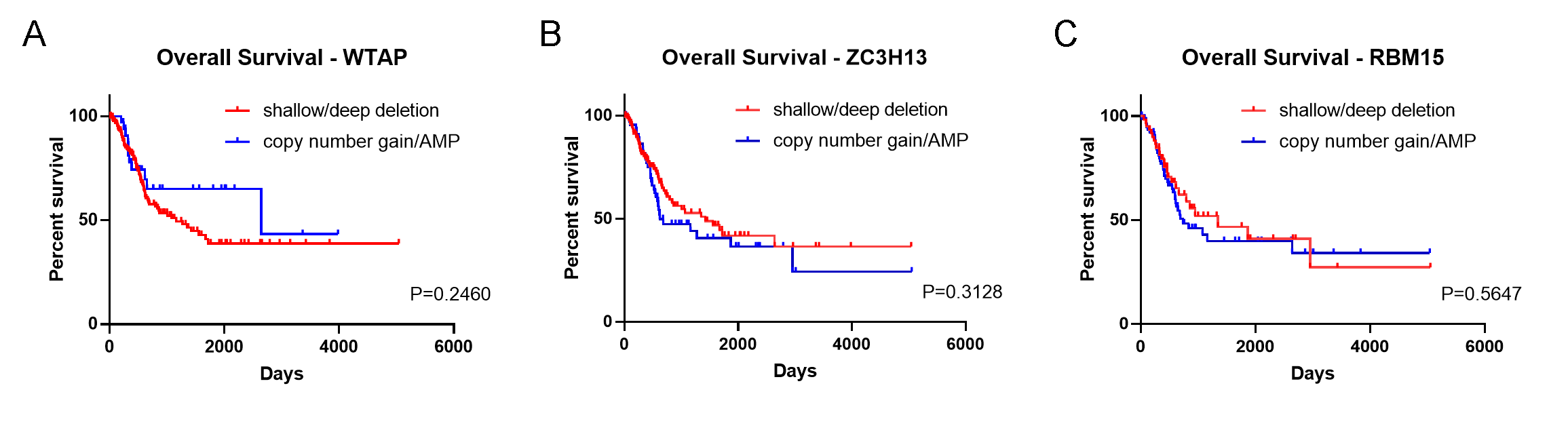

Supplement: Supplementary file 2 — Fig S2 [file CAM4-10-7804-s003.tif]

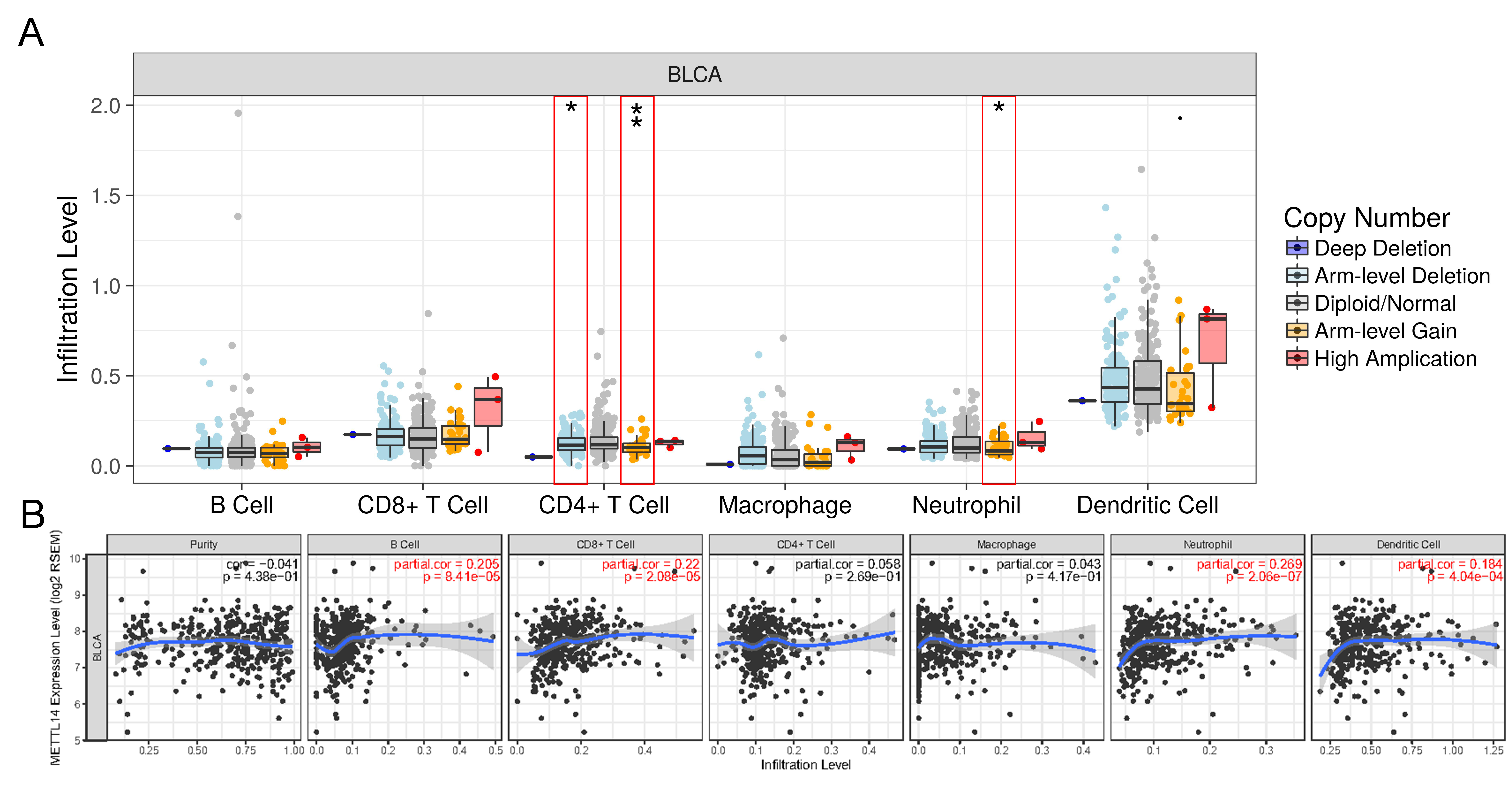

Supplement: Supplementary file 3 — Fig S3 [file CAM4-10-7804-s004.tiff]
