# Supplementary material for: Copy number variation analysis of m6A regulators identified METTL3 as a prognostic and immune‐related biomarker in bladder cancer
Source: Cancer Med. 2021 Oct 20;10(21):7804–15. doi: 10.1002/cam4.3981 (PMC8559456; doi:10.1002/cam4.3981)
Supplement: Supplementary file 4 — Table S1‐S3 [file CAM4-10-7804-s001.docx]

Table S1. The 20 most frequently mutated genes in BLCA generated by previous data.

| **Genes** | **Mutation frequency** |
| --- | --- |
| TP53 | 48.1% |
| TNN | 47.3% |
| MUC16 | 28.4% |
| KMT2D | 28.4% |
| KDM6A | 26.0% |
| ARID1A | 24.5% |
| PIK3CA | 22.1% |
| SYNE1 | 20.4% |
| RYR2 | 18.9% |
| KMT2C | 18.7% |
| HMCN1 | 18.4% |
| RB1 | 17.5% |
| FLG | 16.5% |
| MACF1 | 16.5% |
| EP300 | 15.3% |
| FAT4 | 15.3% |
| FGFR3 | 14.1% |
| ATM | 13.8% |
| STAG2 | 13.8% |
| CSMD3 | 13.8% |

Table S2. Gene sets enrichment of higher *METTL3* mRNA expression level in BLCA patients. Normalized *P* < 0.05 and FDR<0.25 were considered significant.

| **GS DETAILS** | **SIZE** | **ES** | **NES** | **NOM**  ***P*-val** | **FDR**  ***Q*-val** |
| --- | --- | --- | --- | --- | --- |
| HALLMARK_ MITOTIC_SPINDLE | 197 | 0.64 | 2.00 | 0.002 | 0.058 |
| HALLMARK_G2M_CHECKPOINT | 198 | 0.67 | 1.83 | 0.012 | 0.153 |
| HALLMARK_PEROXISOME | 104 | 0.49 | 1.68 | 0.027 | 0.170 |
| HALLMARK_E2F_TARGETS | 200 | 0.67 | 1.71 | 0.045 | 0.176 |
| HALLMARK_PROTEIN_SECRETION | 96 | 0.56 | 1.65 | 0.046 | 0.171 |

Table S3. Gene sets enrichment of higher *METTL14* mRNA expression level in BLCA patients. A total of 33 gene sets were significantly enriched and the top 20 sets were showed. Normalized *P* < 0.05 and FDR<0.25 were considered significant.

| **GS DETAILS** | **SIZE** | **ES** | **NES** | **NOM**  ***P*-val** | **FDR**  ***Q*-val** |
| --- | --- | --- | --- | --- | --- |
| HALLMARK_PI3K_AKT_MTOR_SIGNALING | 104 | 0.70 | 2.26 | <0.001 | 0.001 |
| HALLMARK_PROTEIN_SECRETION | 96 | 0.76 | 2.25 | <0.001 | <0.001 |
| HALLMARK_HEME_METABOLISM | 195 | 0.60 | 2.24 | <0.001 | <0.001 |
| HALLMARK_MITOTIC_SPINDLE | 197 | 0.72 | 2.20 | <0.001 | <0.001 |
| HALLMARK_UV_RESPONSE_DN | 142 | 0.63 | 2.18 | <0.001 | <0.001 |
| HALLMARK_ANDROGEN_RESPONSE | 100 | 0.63 | 2.10 | <0.001 | 0.003 |
| HALLMARK_GLYCOLYSIS | 198 | 0.56 | 2.00 | <0.001 | 0.011 |
| HALLMARK_SPERMATOGENESIS | 134 | 0.55 | 1.96 | <0.001 | 0.009 |
| HALLMARK_PEROXISOME | 104 | 0.55 | 1.93 | <0.001 | 0.009 |
| HALLMARK_DNA_REPAIR | 149 | 0.64 | 1.93 | 0.002 | 0.009 |
| HALLMARK_UNFOLDED_PROTEIN_RESPONSE | 111 | 0.66 | 1.95 | 0.002 | 0.010 |
| HALLMARK_BILE_ACID_METABOLISM | 112 | 0.49 | 1.95 | 0.002 | 0.008 |
| HALLMARK_NOTCH_SIGNALING | 32 | 0.60 | 1.89 | 0.002 | 0.009 |
| HALLMARK_APOPTOSIS | 161 | 0.55 | 1.84 | 0.002 | 0.012 |
| HALLMARK_ESTROGEN_RESPONSE_EARLY | 198 | 0.49 | 1.97 | 0.004 | 0.017 |
| HALLMARK_G2M_CHECKPOINT | 198 | 0.74 | 1.90 | 0.004 | 0.010 |
| HALLMARK_FATTY_ACID_METABOLISM | 158 | 0.53 | 1.86 | 0.004 | 0.011 |
| HALLMARK_WNT_BETA_CATENIN_SIGNALING | 41 | 0.62 | 1.86 | 0.004 | 0.016 |
| HALLMARK_TGF_BETA_SIGNALING | 53 | 0.66 | 1.97 | 0.006 | 0.009 |
| HALLMARK_UV_RESPONSE_UP | 158 | 0.50 | 1.76 | 0.006 | 0.025 |
